# Pigmented Urine

**Published:** 2013-01-23

**Authors:** Rebecca Nunn, Nicholas Chang, Stephen M. Milner, Leigh Ann Price

**Affiliations:** Department of Plastic and Reconstructive Surgery, The Johns Hopkins University School of Medicine, Baltimore, Md

**Figure:**
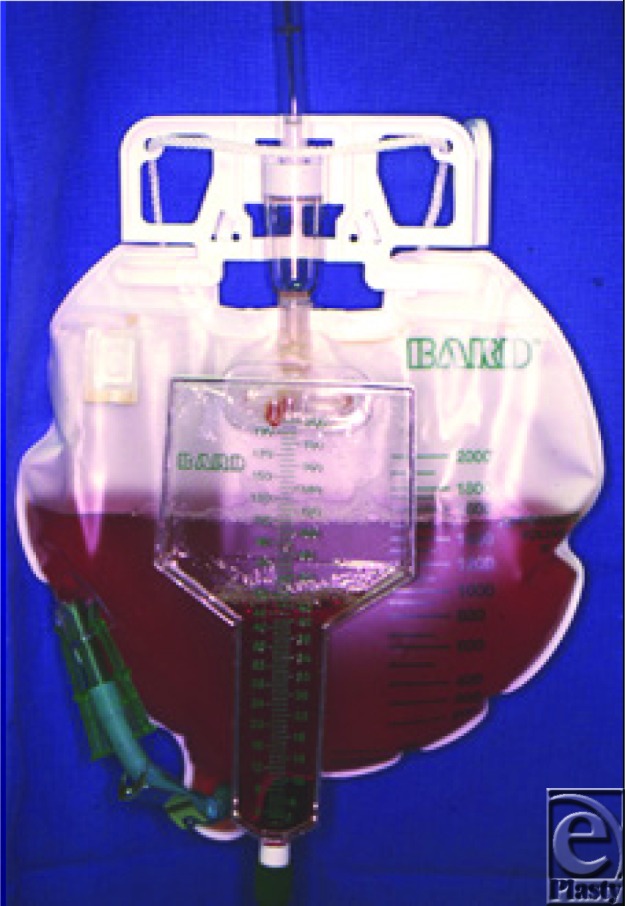


## DESCRIPTION

A previously fit 21-year-old man accidently straddled a 2000 V pylon. His urine, collected 3 hours later, is shown in the figure.

## QUESTIONS

1. **What accounts for the color of the urine?**
2. **What are the complications of this condition?**
3. **Describe the management of the underlying disorder.**

## DISCUSSION

The patient has myoglobinuria, secondary to rhabdomyolysis due to the high-voltage electrical injury. This resulted from rhabdomyolysis, whereby destruction of skeletal muscle has released muscle cell myoglobin, a heme-containing protein, into the circulation.n^4^ The urine is tea-coloured. Under physiological conditions, myoglobin is not present in the urine but is freely filtered by the kidneys and reabsorbed by tubular epithelial cells where it is metabolised.^1^^,^^4^ Myoglobinuria occurs when serum myoglobin levels exceed the renal threshold of 0.5 to 1.5 mg/dL, but the characteristic color is not typically seen until levels reach 100 mg/dL.^1^ Muscle cell damage can occur through a number of mechanisms including heat-induced denaturation of cellular proteins (via the conversion of electrical energy to thermal energy as the current passes through relatively resistance tissues), or the electrical disruption of cell membranes, leading to impaired ion exchange and cellular dysfunction.^2^ In high-voltage electrical injuries, rhabdomyolysis may also occur following the initial trauma (crush injury), or in the context of a later complication such as compartment syndrome.

In the United States, 7% to 10% of all cases of acute kidney injury (AKI) occur after rhabdomyolysis.^1^ Myoglobin is believed to be the central mediator of renal damage. This can be attributed to either the direct generation of highly reactive oxygen species (primarily affecting the proximal tubule) or indirectly due to complex formation with the secreted renal tubular Tamm-Horsfall protein, with subsequent occlusion of the distal tubules. Both processes are exacerbated by acidotic conditions.^1^ In addition, compounding factors, such as hypovolemia and increased catecholamine release, lead to reduced renal perfusion. This contributes to renal ischemia and subsequent damage to the renal parenchyma.^1^ Additional complications of rhabdomyolysis include electrolyte disturbances (hyperkalemia, hyperphosphatemia, hyperuricemia and hypocalcemia), metabolic acidosis, hypovolemia, compartment syndrome, arrhythmias, disseminated intravascular coagulation, and hepatic dysfunction.^4^

The appearance of tea-coloured urine may be the first clinical sign of rhabdomyolysis. This is confirmed by elevated plasma creatine kinase levels.^3^ Prompt, aggressive fluid therapy is key to preventing AKI. This dilutes myoglobin levels, reducing the accumulation of precipitants and toxic by-products in the renal tubules.^1^^,^^4^ Alkalinization of the urine is widely believed to reduce myoglobin-induced damage by reversing acidotic conditions.^4^ Conventionally sodium bicarbonate has been used to achieve alkalinization, although there is little evidence to support the efficacy of sodium bicarbonate administration over aggressive fluid resuscitation with crystalloid.^1^^,^^3^ Mannitol, an osmotic diuretic, is also often included in treatment algorithms despite little evidence to support its use.^1^^,^^4^ The theoretical advantages of mannitol in the prevention of rhabdomyolysis-associated AKI is that it acts to reduce tissue edema while increasing the intravascular volume, increases the renal clearance of toxic substances, and also acts as a free radical scavenger.^1^ In addition, electrolyte disturbances, particularly hyperkalemia, should be carefully monitored and may require treatment. If these measures are unsuccessful, renal-replacement therapy may be required, and permanent renal damage may ensue.^1^ Potential future treatments, including antioxidants and free radical scavengers such as vitamins A and E, are currently being investigated for their role in the clinical management of myoglobinuria-associated rhabdomyolysis.^1^

## References

[1] Bosch X, Poch E, Grau JM (2009). Rhabdomyolyisis and acute kidney injury. N Eng J Med.

[2] Handschin AE, Vetter S, Jung FJ, Guggenheim M, Kunzi W, Giovanoli P (2009). A Case-matched controlled study on high-voltage electrical injuries vs thermal burns. J Burn Care Res.

[3] Huerta-Alardin AL, Varon J, Marik PE (2005). Bench-to-bedside review: rhabdomyolysis—an overview for clinicians. Crit Care.

[4] Khan FY (2009). Rhabdomyolysis: a review of the literature. Neth J Med.

